# Atractylenolide-I Protects Human SH-SY5Y Cells from 1-Methyl-4-Phenylpyridinium-Induced Apoptotic Cell Death

**DOI:** 10.3390/ijms18051012

**Published:** 2017-05-08

**Authors:** Sandeep Vasant More, Dong-Kug Choi

**Affiliations:** Department of Biotechnology, College of Biomedical and Health Science, Konkuk University, Chungju 380-701, Korea; sandeepbcp@gmail.com

**Keywords:** atractylenolide-I, apoptosis, MPP^+^, neuroprotection, Parkinson’s disease

## Abstract

Oxidative stress and apoptosis are the major mechanisms that induce dopaminergic cell death. Our study investigates the protective effects of atractylenolide-I (ATR-I) on 1-methyl-4-phenylpyridinium (MPP^+^)-induced cytotoxicity in human dopaminergic SH-SY5Y cells, as well as its underlying mechanism. Our experimental data indicates that ATR-I significantly inhibits the loss of cell viability induced by MPP^+^ in SH-SY5Y cells. To further unravel the mechanism, we examined the effect of ATR-I on MPP^+^-induced apoptotic cell death characterized by an increase in the Bax/Bcl-2 mRNA ratio, the release of cytochrome-c, and the activation of caspase-3 leading to elevated levels of cleaved poly(ADP-ribose) polymerase (PARP) resulting in SH-SY5Y cell death. Our results demonstrated that ATR-I decreases the level of pro-apoptotic proteins induced by MPP^+^ and also restored Bax/Bcl-2 mRNA levels, which are critical for inducing apoptosis. In addition, ATR-I demonstrated a significant increase in the protein expression of heme-oxygenase in MPP^+^-treated SH-SY5Y cells. These results suggest that the pharmacological effect of ATR-I may be, at least in part, caused by the reduction in pro-apoptotic signals and also by induction of anti-oxidant protein.

## 1. Introduction

A new flow of information indicates that oxidative stress induced by reactive oxygen species (ROS) is involved in selective nigral cell degeneration [1]. Postmortem studies of Parkinson’s disease (PD) patients have provided evidence for chemical changes that are indicative of oxidative stress in the substantia nigra (SN) [2,3,4]. Neurons are highly sensitive to ROS, which are well-known triggers of programmed cell death. It is well established that human neurodegenerative pathologies, including stroke, Alzheimer’s disease, and PD, involves several signaling pathways [5] fueled by oxidative stress leading to neuronal loss. The intrinsic mitochondrial signaling pathway is also involved in executing cell death initiated by 1-methyl-4-phenyl-2,3,6-tetrahydropyridine (MPTP) [6].

Experimental use of 1-methyl-4-phenylpyridinium (MPP^+^) in animal and cellular models is very well known to induce symptoms closely resembling PD [7]. MPP^+^ is the active metabolite of MPTP, which accumulates within dopaminergic neurons up to millimolar concentration in the mitochondria, and selectively inhibits nicotinamide adenine dinucleotide coenzyme (NADH Co) Q1 reductase (complex I) of the mitochondrial electron transport chain [8]. Inhibition of complex-I increases the shunting of electrons through complex-II, which generates 5–7 times more ROS [9]. A decrease in mitochondrial complex-I activity primes Bax-dependent neuronal apoptosis through mitochondrial oxidative damage [10]. It is also documented that neuronal cell death induced by MPP^+^ is mediated by the opening of the mitochondrial permeability transition pore and the collapse of the mitochondrial membrane potential [11]. Additionally, the members of Bcl-2 family also play a significant role in MPP^+^-induced apoptotic cell death [12]. As reported, the death of dopaminergic neurons by apoptosis might be initiated by oxidative stress and neuroinflammation [13,14]. Apoptosis induced by oxidative stress is associated with the release of cytochrome c, activation of caspases, and cleavage of poly(ADP-ribose) polymerase (PARP) [15,16,17]. Therefore, inhibition of pro-apoptotic signaling molecules, along with ROS, may have therapeutic benefits for protecting dopaminergic neurons in PD.

Hence, there is a need to develop new protective agents that can avoid the succession of such neuronal apoptosis. Extracts from medicinal plants, secondary metabolites, and their bioactive ingredients have conventionally been used to treat numerous neurodegenerative diseases [18]. Information from various reports have documented that several oriental herbs from plants, or nutrients from foods, have protective activity against apoptosis and are potential therapeutic agents [19]. Lately, there has been a growing interest in research concerning natural products due to the failure of substitute drug discovery methods to deliver many leading compounds in key therapeutic areas [20]. Hence, the industry in the pharmaceutical segment is focused on developing new drugs based on natural sources through the examination of leads from the traditional systems of medicine [20]. In our current study, we have used atractylenolide-I (ATR-I); an active principle obtained from rhizomes of *Atractylodes macrocephala* belonging to the *Asteraceae* family. Pharmacological studies have designated numerous activities of ATR-I in biological systems, such as gastrointestinal inhibitory effects [21], anti-oxidant activity [22], anti-inflammatory activity and anti-cancer activity [23,24]. Based on those reports of ATR-I we hypothesized that ATR-I might be a neuroprotective agent in MPP^+^-induced neuronal damage by inhibiting oxidative stress and apoptotic cell death. We, therefore, explored the therapeutic potential of ATR-I, and explored possible mechanisms in the MPP^+^-induced PD model in SH-SY5Y cells.

## 2. Results

### 2.1. Effects of Atractylenolide-I (ATR-I) on Cytotoxicity Induced by 1-Methyl-4-Phenylpyridinium (MPP^+^) in SH-SY5Y Cells

**Figure 1:**
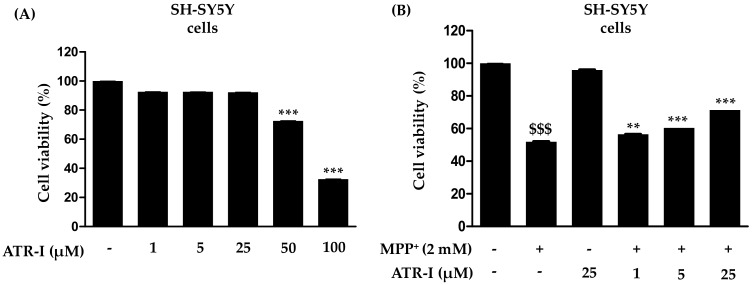
(**A**,**B**) Effects of atractylenolide-I (ATR-I) on cell viability in SH-SY5Y cells intoxicated with or without 1-methyl-4-phenylpyridinium (MPP^+^). The viability of cells was performed as mentioned in the “Material and Method” section. *** *p* < 0.001 vs. vehicle group. ^$$$^ *p* < 0.001 vs. vehicle group, and *** *p* < 0.001, ** *p* < 0.01 vs. MPP^+^-treated group.

To investigate whether ATR-I causes mortality in SH-SY5Y cells, they were incubated with various concentrations of ATR-I (1, 5, 25, 50 and 100 µM) for 24 h (Figure 1A). Our results indicated that ATR-I (1, 5, 25 µM) did not show any significant cytotoxicity in SH-SY5Y cells for 24 h. While the higher doses (50 and 100 µM) were observed to significantly decrease cell viability. Furthermore, we tested the effect of ATR-I (1, 5 and 25 µM) in combination with 2 mM MPP^+^. As illustrated in Figure 1B, MPP^+^-induced a significant decrease (48%) in cell viability as compared to the vehicle group. However, pre-incubation with ATR-I (1, 5 and 25 µM) prevented cells from MPP^+^-induced cell damage by dose-dependently restoring cell viability to 56.50%, 60.49%, and 71.49% in comparison to MPP^+^ group.

### 2.2. ATR-I Abates Bax, Bcl-2 Ratios and Upregulates Heme Oxygenase (HO-1) mRNA and Protein Expression in MPP^+^-Intoxicated SH-SY5Y Cells

**Figure 2:**
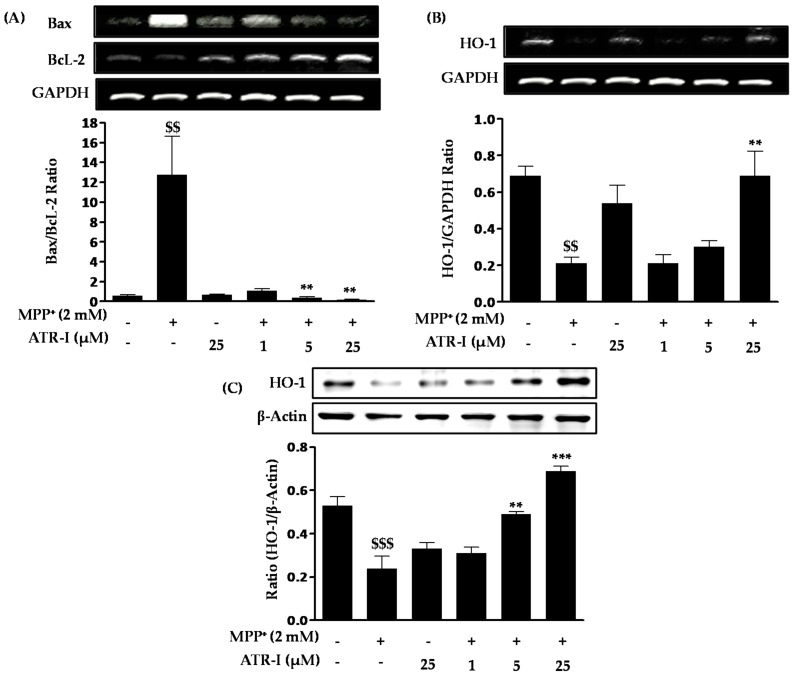
(**A**) Effects of ATR-I on the Bax/Bcl-2 mRNA ratio in MPP^+^-stimulated SH-SY5Y cells. The levels of (**B**) HO-1 mRNA and (**C**) HO-1 protein expression were quantitated by densitometric analysis. Quantification data are shown in the lower panel. Glyceraldehyde-3-phosphate dehydrogenase (GAPDH) and β-actin were used as internal controls. ^$$$^ *p* < 0.001 and ^$$^ *p* < 0.01 vs. the vehicle group; *** *p* < 0.001 and ** *p* < 0.01 vs. the MPP^+^-treated group.

As shown in Figure 2A, exposure to MPP^+^ significantly increases Bax mRNA expression (nine-fold) in comparison to the control group, a finding which is consistent with previous reports [25,26], while pre-treatment with ATR-I (1 µM (39%), 5 (15%) µM and 25 (12%) µM) dose-dependently decreases Bax mRNA expression in comparison to MPP^+^-intoxicated cells. In contrast to Bax, the levels of Bcl-2 mRNA decreased (2-fold) in the MPP^+^-treated group as compared to the control group. These levels were dose-dependently increased after ATR-I treatment (1 (3.7-fold), 5 (4.57-fold), and 25 (7.2-fold) µM) in comparison to MPP^+^-intoxicated cells. The Bax/Bcl-2 ratio in cells exposed to 2 mM MPP^+^ was 12-fold higher than the control group, while in cells pre-treated with 1, 5 and 25 µM ATR-I, the ratio decreased (11, 33 and 67-fold) in a dose-dependent fashion, suggesting that ATR-I treatment shifted the balance between pro- and anti-apoptotic members towards cell survival (Figure 2A). ATR-I treatment alone did not significantly alter the Bax/Bcl-2 ratio. Exposure to 2 mM MPP^+^ was found to decrease the mRNA levels (3.2-fold) of heme oxygenase (HO-1) as compared to control group in SH-SY5Y cells. However, this decrease was reversed by pre-treatment with ATR-I (25 µM) by three-fold in comparison to MPP^+^-intoxicated cells, respectively (Figure 2B). On the other hand, our data in Figure 2C, correlates with the dose-dependent rise (1 (1.2-fold), 5 (two-fold), and 25 (three-fold) µM) in the protein expression profile of HO-1 by ATR-I in MPP^+^-stimulated SH-SY5Y cells. Thus, induction of HO-1 expression by ATR-1 suggests a role for an antioxidant mechanism in the protection of neuronal cells against MPP^+^-dependent cytotoxicity.

### 2.3. ATR-I Pre-Treatment Mitigates MPP^+^-Induced Protein Expression of p53 and Cytochrome-c in SH-SY5Y Cells

**Figure 3:**
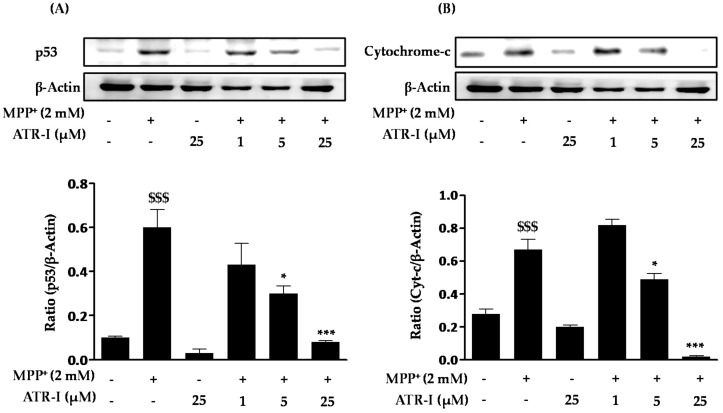
Effect of ATR-I on the MPP^+^-induced p53 and cytochrome-c release in SH-SY5Y cells. SH-SY5Y cells were subjected to Western blotting with specific antibodies for p53 (**A**) and cytochrome-c (**B**). Quantification of the relative expression of p53 and cytochrome-c is provided in the bar graph. β-actin was used as an internal control. ^$$$^ *p* < 0.001 vs. the vehicle group; *** *p* < 0.001 and * *p* < 0.05 vs. the MPP^+^-treated group.

As shown in Figure 3A, MPP^+^ treatment resulted in a significant increase (six-fold) in the levels of p53 in SH-SY5Y cells as compared to the control. Pre-treatment with various doses of ATR-I (1, (1.5-fold), 5 (2-fold), and 25 (7.5-fold) µM) dose-dependently decreased protein levels of p53 in comparison to MPP^+^-intoxicated cells. Additionally, as seen in Figure 3B, exposure of MPP^+^ to SH-SY5Y cells significantly increased the levels of cytochrome-c (2.5-fold) as compared to the control group. Pre-treatment of ATR-I (1 (1.2-fold), 5 (1.3-fold), and 25 (33-fold) µM) was found to reduce the levels of cytochrome-c in comparison to MPP^+^-intoxicated cells.

### 2.4. ATR-I Pre-Treatment Prevents MPP^+^-Induced Induction of Caspase-3 and Cleaved Poly(ADP-Ribose) Polymerase (PARP) in SH-SY5Y Cells

**Figure 4:**
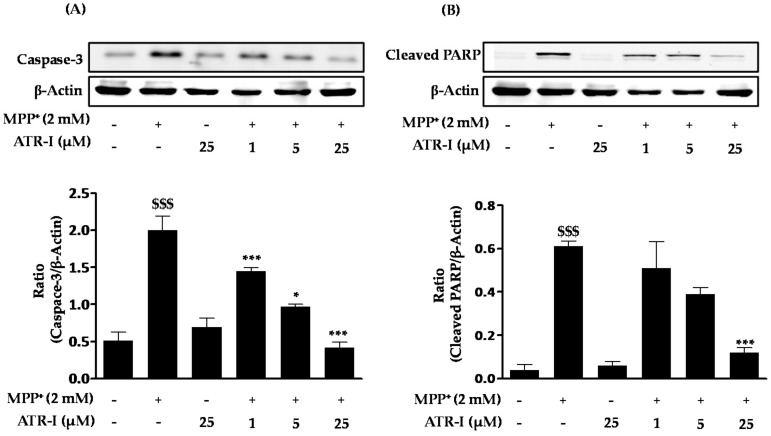
Effect of ATR-I on MPP^+^-induced expression of caspase-3 and cleaved poly(ADP-ribose) polymerase (PARP) in SH-SY5Y cells. SH-SY5Y cells were tagged with specific antibodies for caspase-3 (**A**) and cleaved PARP (**B**). Quantification of relative expression of caspase-3 and cleaved PARP is provided in the bar graph. β-actin was used as an internal control. ^$$$^ *p* < 0.001 vs. the vehicle group; *** *p* < 0.001 and * *p* < 0.05 vs. the MPP^+^-treated group.

Exposure of MPP^+^ to SH-SY5Y cells significantly increased (3-fold) the protein levels of caspase-3 (Figure 4A) as compared to the control group. However, pre-treatment with ATR-I was successful in dose-dependently reversing (1 (1.4-fold), 5 (two-fold), and 25 (4.7-fold) µM) the increased levels caspase-3 in comparison to MPP^+^ intoxicated cells. Furthermore, MPP^+^ was found to markedly increase (15-fold) the protein levels of cleaved PARP (Figure 4B) as compared to the control group. However, only the highest dose (25 µM) of ATR-I decreased the levels of cleaved PARP (five-fold) as compared to the MPP^+^ group. Lower doses of ATR-I (1 and 5 µM) were not observed to decrease the levels of cleaved PARP (1.1- and 1.5-fold).

### 2.5. Protective Role of HO-1 in MPP^+^-Intoxicated SH-SY5Y Cells

**Figure 5:**
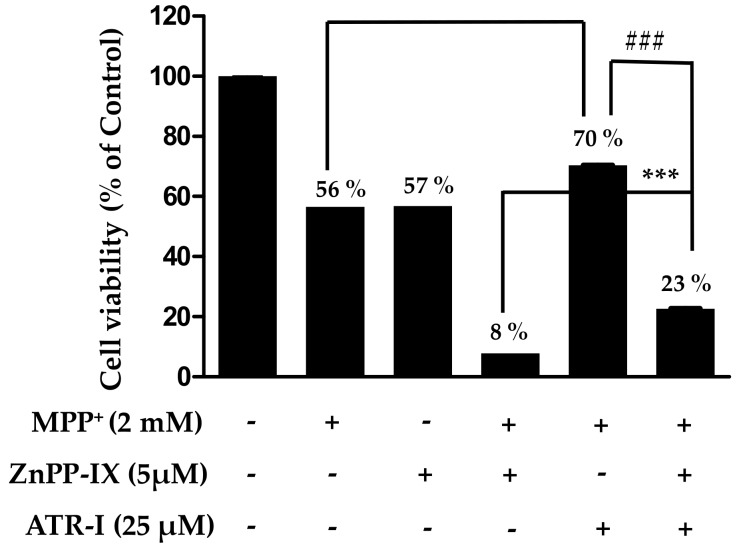
SH-SY5Y cells were pre-incubated with or without zinc protoporphyrin-IX (ZnPP-IX ) (5 µM) for 2 h before treating them with ATR (25 µM) for another 2 h. Cells were further exposed to 2 mM MPP^+^ for 24 h. Cell viability was measured by MTT assay. ^$$$^ *p* < 0.001 vs. the MPP^+^ group, ^###^ *p* < 0.001 vs. the MPP^+^ + ATR-I group and *** *p* < 0.001 vs. the ZnPP-IX + MPP^+^-treated group.

As presented in Figure 5, sole treatment with either MPP^+^ or zinc protoporphyrin-IX (ZnPP-IX) reduces the cell viability (56–57%) to a significant extent in comparison to the vehicle group, while a combination of ZnPP-IX (5 µM) and 2 mM MPP^+^ aggravates the cytotoxicity (8%) in SH-SY5Y cells in comparison to the vehicle group. Additionally, there is a significant difference in cell viability between MPP^+^ relative to MPP^+^/ATR-I and MPP^+^/ATR-I relative to MPP^+^/ZnPP-IX/ATR-I. Pre-treatment with ATR-I (25 µM) to cells exposed with ZnPP-IX (5 µM) + 2 mM MPP^+^ was found to increase (three-fold) the cell viability to a significant extent in comparison to the ZnPP-IX + MPP^+^-treated group. This data suggests that HO-1 plays an important role in protecting the SH-SY5Y cells against MPP^+^-induced cell death.

## 3. Discussion

It is well accepted that many neurodegenerative diseases are mediated, at least in part, by apoptosis and oxidative stress. A variety of mechanisms that lead to apoptosis have been extensively characterized in a wide range of neuronal types. Mitochondria are a critical starting point for a multitude of apoptotic insults initiating a process described as the intrinsic death pathway [27]. Conflicting evidence has been presented as to which particular steps are essential for initiation of intrinsic neuronal apoptosis [28]. MPP^+^-treated dopaminergic human SH-SY5Y neuroblastoma cells are an established model to study neuronal apoptosis that has been widely used to study the neurodegenerative events that occur in PD. In our preliminary experiments, we found 2 mM MPP^+^ and an incubation time of 24 h were the optimal concentration and time for the induction of deleterious effects on cell viability of SH-SY5Y cells, which was in agreement with previous research [29]. In the present study, using undifferentiated SH-SY5Y cells, which are more susceptible to MPP^+^-induced cell death [30], we demonstrated that pre-treatment with ATR-I protected SH-SY5Y cells against MPP^+^-induced cytotoxicity by multiple lines of evidence.

There is increasing evidence that oxidative stress plays a key role in the pathogenesis of PD [31]. MPP^+^ has been shown to cause neuronal cell death by inducing mitochondrial dysfunction and increasing ROS production [32]. ROS over-production can severely disrupt the mitochondrial membrane potential, resulting in the release of pro-apoptosis factors and neuronal cell death. HO-1 is an anti-oxidant and anti-apoptotic protein that is induced against oxidative stress [33]. Induction of HO-1 has been demonstrated to attenuate glutamate-induced apoptosis [34]. Hence, to unravel the protective role of HO-1, we have used ZnPP-IX (a specific inhibitor of HO-1) in our study. Only treatment with 25 µM ATR-I was not observed to significantly increase mRNA and protein expression of HO-1 in SH-SY5Y cells. However, a combination of ATR-I with MPP^+^ in SH-SY5Y cells significantly induced mRNA (ATR-I at 25 µM) and protein expression (ATR-I at 5 and 25 µM) of HO-1 in SH-SY5Y cells. Furthermore, ZnPP-IX, was observed to decrease cell viability comparable to that elicited by MPP^+^-treatment. Combination treatment of ZnPP-IX + MPP^+^ decreased the cell viability to a greater extent as compared to the MPP^+^ group, per se. Pre-treatment with ATR-I was found to significantly increase the cell viability in the ZnPP + MPP^+^ co-exposed group. This data suggests that inducing HO-1 levels increases cellular viability and, thus, confers protection to SH-SY5Y cells. Our results are in agreement with earlier reports wherein induction of HO-1 in cells has been shown to confer protection to cells against oxidative stress [35,36,37].

ROS have multiple effects on cell function, depending on the amount and subcellular location of the ROS generated [38]. Some studies have reported that ROS are involved in the apoptotic mechanism of MPP^+^-mediated neurotoxicity [39,40]. p53 is a key modulator of cellular stress responses and is thought to mediate apoptosis in several neurodegenerative diseases, including PD [41]. Furthermore, it has been reported to play a prominent role in mediating cell death in several experimental models of PD [42,43,44]. Since SH-SY5Y cells express wild-type p53 [45], they are suitable for investigating regulation and functions of this protein. p53 is a tumor suppressor protein that is present at low concentrations in normal cells. However, when cells are subjected to stress and injury, protein levels of p53 are increased, leading to apoptosis [46]. It has been reported that MPP^+^ induces apoptosis through the activation of the p53 pathway [43]. To determine whether ATR-I pre-treatment had any effect on the levels of p53, Western blot analysis of total lysates was performed. Our results indicated that pre-treatment with ATR-I significantly diminished the protein levels of p53 in MPP^+^ stimulated in SH-SY5Y cells. Bax is a proapoptotic member of the Bcl-2 family and is also the target gene for p53, which is closely associated to mediate cell death in PD [47,48]. On the other hand, the Bcl-2 family of proteins is involved in positive and negative regulation of apoptotic cell death [49]. O’Malley et al. [50] reported that the over-expression of Bcl-2 attenuates MPP^+^-induced cell death. Bcl-2 and Bax collectively control the permeability of mitochondrial transition pore that results in apoptosis [44,51,52]. Cell survival in the early phases of the apoptotic cascade depends mostly on the balance between the pro- and anti-apoptotic proteins of the Bcl-2 family. In this regard, the Bax/Bcl-2 mRNA ratio may be a better predictor of apoptotic fate than the absolute concentrations of either Bax or Bcl-2 alone [53]. In our study, exposure to MPP^+^ led to significant upregulation of Bax mRNA expression and diminished Bcl-2 mRNA expression in SH-SY5Y cells. Consequently, the ratio of the Bax/Bcl-2 mRNA increased significantly upon treatment with MPP^+^. However, pre-treatment with ATR-I significantly decreased the Bax/Bcl-2 mRNA ratio in MPP^+^-stimulated SH-SY5Y cells.

MPP^+^-induced ROS and Bax have been documented to affect the mitochondrial permeability pore and, thereby, release cytochrome-c in the cytoplasm which further combines with apoptosis activating factor (Apaf-1) and procaspase-9 to further activate caspase-3 [17,54]. It has been reported that caspase-3 acts as an apoptotic executor in both pathways by activating DNA fragmentation to cause apoptotic cell death and decrease in caspase-3 activity is associated with a decrease in the Bax/Bcl-2 ratio [55]. In our study, exposure of MPP^+^ to SH-SY5Y cells considerably increased protein expression of cytochrome-c and caspase-3. Our results are in accordance with an earlier report by Doo et al. wherein suppression of cytochrome-c and caspase-3 expression provide protective effects against MPP^+^-induced apoptosis in SH-SY5Y cells. It has also been reported that activation of caspase-3 leads to the cleavage of a number of proteins, including PARP [16,56]. PARP, one of downstream targets of caspase-3, is an abundant nuclear enzyme that normally takes part in DNA repair, but extensive PARP activation can promote cell death [57]. In this study, ATR-I pre-treatment was found to reduce the cleavage of PARP in MPP^+^-intoxicated SH-SY5Y cells. These observations imply that ATR-I may modulate the expression of Bax/Bcl-2 mRNA in response to MPP^+^ treatment, regulating a succession of mitochondria-mediated downstream molecular events including the caspase-3 activation and PARP proteolysis.

## 4. Materials and Methods

### 4.1. Reagents and Antibodies

ATR-I (purity ≥ 98%) was purchased from BaoJi Herbest Bio-Tech Co., Ltd. (Baoji, China). MPP^+^, 3-(3,4-dimehylthiazol-2-yl)-2,5-diphenyl-tetrazolium bromide (MTT), and ZnPP-IX were obtained from Sigma-Aldrich (St. Louis, MO, USA). Six-well and 96-well tissue culture plates and 100 mm culture dishes were purchased from Nunc Inc. (North Aurora Road, Naperville, IL, USA). Dulbecco’s modified Eagle’s medium (DMEM/F12 + Glutamax) and fetal bovine serum (FBS) were obtained from Gibco-BRL Technologies (Gaithersburg, MD, USA). The antibody for p53 and cytochrome-c were procured from Santa Cruz Biotechnology (Santa Cruz, CA, USA). Antibodies for caspase-3, cleaved PARP, and β-actin were obtained from Cell Signaling Co. (Boston, MA, USA). The antibody for HO-1 was obtained from Enzo Life Sciences (Farmingdale, NY, USA). All other chemicals used in this study were of analytical grade and were obtained, unless otherwise noted, from Sigma-Aldrich.

### 4.2. Cell Culture and Treatments

The human dopaminergic neuroblastoma cell line, SH-SY5Y, was purchased from American Type Culture Collection (ATCC) (Manassas, VA, USA). SH-SY5Y cells were cultured in DMEM/F12 + Glutamax supplemented with 10% (*v*/*v*) inactivated fetal bovine serum, and 100 U/mL penicillin/streptomycin. The cells were maintained at 37 °C in a 5% CO_2_ and 95% humidified air incubator for the indicated time. ATR-I was dissolved in dimethyl sulfoxide (DMSO). The control group was treated with only DMSO (vehicle group).

### 4.3. Measurement of Cell Viability

Viability of cultured cells was measured by the reduction of MTT to formazan [58]. SH-SY5Y cells (2.5 × 10^4^ cells/mL) were pre-treated with different doses of ATR-I (1, 5, 25, 50, and 100 µM) for 2 h in 96-well plates and then stimulated with or without MPP^+^ (2 mM) for 24 h. After the treatment for 24 h, the medium was removed and the cells were incubated with 0.5 mg/mL of MTT solution for 4 h at 37 °C. The supernatant was carefully removed, and the formed formazan crystals were dissolved in DMSO and the absorbance at 550 nm was measured using a microplate reader. Cell viability was expressed as a percentage of the control (untreated cells).

### 4.4. Isolation of Total RNA and Reverse Transcription Polymerase Chain Reaction (RT-PCR)

SH-SY5Y cells seeded in six-well plates (50 × 10^4^ cells/mL) were pretreated with ATR-I (1, 5, and 25 µM) for 2 h and then exposed with or without 2 mM MPP^+^ for 6 h. Total RNA was isolated by extraction with TRIzol (Invitrogen) (Burlington, ON, Canada). For the reverse transcription-polymerase chain reaction (RT-PCR), 2.5 µg of total RNA from each group was reverse-transcribed using a First-Strand cDNA synthesis kit (Invitrogen). Then, cDNA was amplified by PCR using specific primers, as mentioned previously [59]. The PCR was performed using the above-prepared cDNA as a template for respective targets. The following primers were used for PCR: Bax: F-5′-CACCAAGGTGCCGGAACTGA-3′ and R-5′-AATGCCCATGTCCCCCAAT-3′; BcL-2: F-5′-ACGACTTCTCCCGCCGCTAC-3′ and R-5′-CCCAGCCTCCGTTATCCTGG-3′; HO-1: F-5′-CACGCCTACACCCGCTACCT-3′ and R-5′-TCTGTCACCCTGTGCTTGAC-3′; and GAPDH: F-5′-GTCAGTGGTGGACCTGACCT-3′ and F-5′-GTCAGTGGTGGACCTGACCT-3′. PCR products were analyzed on 1% agarose gels stained with ethidium bromide, and bands were visualized by UV light.

### 4.5. Sodium Dodecyl Sulfate-Polyacrylamide Gel Electrophoresis (SDS-PAGE) and Western Blot Analysis

SH-SY5Y cells (50 × 10^4^ cells/mL) cultured in six-well plates were pretreated when the cell confluency was 75% without or with ATR-I (1, 5, and 25 µM) for 2 h, followed by treatment with LPS for 18 h. Preparation of cell lysates and electrophoresis and immunoblotting procedures followed a previous report [59]. PVDF membranes were labeled with respective antibodies by incubating them overnight with anti-p53 (1:1000), anti-cytochrome-c (1:1000), anti-caspase-3 (1:1000), anti-cleaved PARP (1:1000), anti-TH (1:1000), anti-HO-1 (1:1000), and anti-β-actin (1:2000) antibodies, followed by a 1 h incubation with horseradish peroxidase-conjugated secondary antibodies (1:1000–1:5000) (Cell Signaling Technology) (Danvers, MA, USA) and (Santa Cruz biotechnology). The optical densities of the antibody-specific bands were analyzed with a Luminescent Image Analyzer, LAS-3000 (Fuji, Tokyo, Japan).

### 4.6. Statistical Analyses

Statistical analyses were performed using GraphPad software, version 5 (GraphPad, La Jolla, CA, USA). Data are expressed as means ± standard error (SEM) of at least three independent experiments. Significant differences between the groups were determined using a one-way analysis of variance (ANOVA) followed by Tukey’s post hoc analysis. *p*-values < 0.05 were considered statistically significant.

## 5. Conclusions

**Figure 6:**
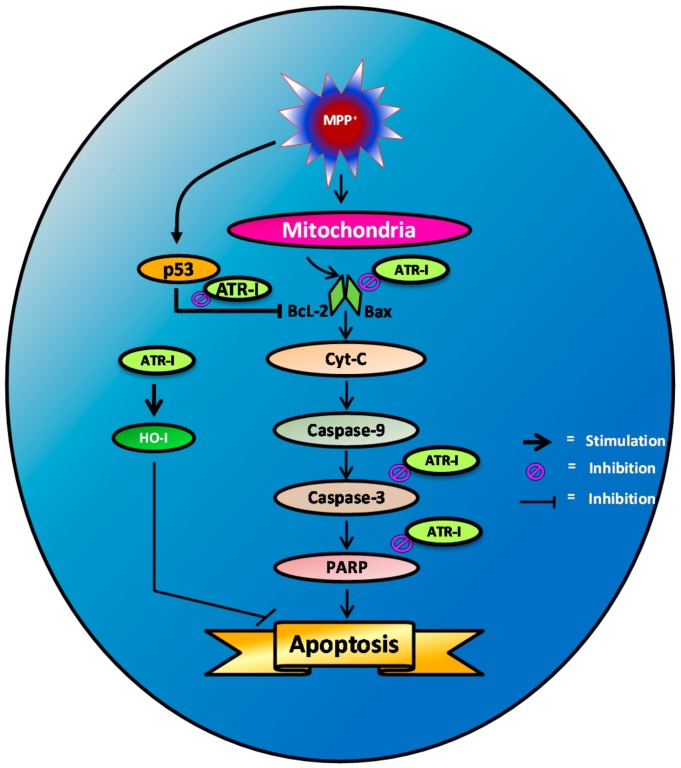
Scheme representing MPP^+^-induced apoptotic signaling

In summary (Figure 6), our results show that pre-treatment of SH-SY5Y cells with ATR-I significantly decreases MPP^+^-induced apoptotic cell death. This anti-apoptotic effect is a result of diminished caspase-3 and cleaved PARP release that subsequently abates pro-apoptotic signaling in the mitochondria, thereby decreasing cell death. Additionally, ATR-I by its anti-oxidant effect, evident by the upregulation of HO-1 release, might also contribute to the cytoprotective effect in SH-SY5Y cells. Our study reports that ATR-I could ameliorate MPP^+^-induced oxidative stress and apoptosis in SH-SY5Y cells and exert its neuroprotective activity partly by decreasing oxidative stress and by inhibiting pro-apoptotic signals.

## Abbreviations

ATR-I	Atractylenolide-I
Apaf	Apoptotic protease activating factor 1
DMSO	Dimethyl sulfoxide
HO-1	Oxygenase-1
MPP	1-methyl-4-phenylpyridinium
MPTP	1-methyl-4-phenyl-1,2,3,6-tetrahydropyridine
PD	Parkinson’s disease
PARP	(ADP-ribose) polymerase
PCD	Programmed cell death
ROS	Reactive oxygen species
SN	Substantia nigra
ZnPP	Zinc protoporphyrin
